# Survey on Main Drive Methods Used in Humanoid Robotic Upper Limbs

**DOI:** 10.34133/2021/9817487

**Published:** 2021-06-15

**Authors:** Yiwei Wang, Wenyang Li, Shunta Togo, Hiroshi Yokoi, Yinlai Jiang

**Affiliations:** ^1^Department of Mechanical Engineering and Intelligent Systems, The University of Electro-Communications, Japan; ^2^Center for Neuroscience and Biomedical Engineering, The University of Electro-Communications, Tokyo, Japan; ^3^Beijing Advanced Innovation Center for Intelligent Robots and System, Beijing Institute of Technology, Beijing, China

## Abstract

Humanoid robotic upper limbs including the robotic hand and robotic arm are widely studied as the important parts of a humanoid robot. A robotic upper limb with light weight and high output can perform more tasks. The drive system is one of the main factors affecting the weight and output of the robotic upper limb, and therefore, the main purpose of this study is to compare and analyze the effects of the different drive methods on the overall structure. In this paper, we first introduce the advantages and disadvantages of the main drive methods such as tendon, gear, link, fluid (hydraulic and pneumatic), belt, chain, and screw drives. The design of the drive system is an essential factor to allow the humanoid robotic upper limb to exhibit the structural features and functions of the human upper limb. Therefore, the specific applications of each drive method on the humanoid robotic limbs are illustrated and briefly analyzed. Meanwhile, we compared the differences in the weight and payload (or grasping force) of the robotic hands and robotic arms with different drive methods. The results showed that the tendon drive system is easier to achieve light weight due to its simple structure, while the gear drive system can achieve a larger torque ratio, which results in a larger output torque. Further, the weight of the actuator accounts for a larger proportion of the total weight, and a reasonable external placement of the actuator is also beneficial to achieve light weight.

## 1. Introduction

Humanoid robots are designed to mimic the appearance and behavior of humans and to perform specific tasks in conjunction with or instead of humans [1]. Many studies have been conducted on humanoid robots, and the humanoid robotic upper limb has been the preferred choice for many researchers [2–4]. Currently, many robotic hands and arms have been commercialized, and they are used in daily life activities and production.

The upper limb has a large number of bones and joints, and many of these joints move independently [5, 6]. Therefore, to design robotic upper limbs, many design requirements need to be considered to achieve a functionality similar to that of the human upper limb. For example, for robotic hands, the main considerations are the number of fingers, size, weight, degrees of freedom (DOFs), grasping force, and fingertip force, whereas for robotic arms, they are length, weight, DOFs, and payload [7, 8]. Puig et al. [9] proposed a five-step design methodology for a multifinger robotic hand: problem definition, concept design, preliminary design, detail design, and design communication, where the concept design phase is considered to address three main elements: actuation, sensors, and control system. In this study, we focus on the design of the actuation, compare and analyze the effect of actuation design on the overall structure, and identify how to choose the appropriate actuation form.

Actuation comprises the actuator and the drive system, where the actuator provides the motion and power output, while the drive system transfers the motion and power to the required position. Actuators are categorized into electronic, pneumatic, and hydraulic actuators, among which the electronic actuators are divided into DC, AC, and stepper motors [10]. Humanoid robotic upper limbs mainly use electronic actuators, and the main drive methods can be categorized into tendon, gear, link, fluid (hydraulic and pneumatic), belt, chain, and screw drives. These different drive methods have different weights, sizes, transmission distances, stiffness, transmission accuracies, and transmission efficiencies, where the achievable transmission ratio also has a large difference. Thus, it is necessary to choose and combine the appropriate drive methods based on design requirements. Usually, a hybrid drive system consisting of multiple drive methods is more difficult to implement than that consisting of a single drive method; however, multiple drive methods can help compensate for each other's limitations.

Mechanical transmission can be categorized into friction and engagement drives based on the principle of transmission [11]. On the one hand, in a friction drive, power and motion are transferred via friction, such as in a belt drive. Although friction transmission cannot be used for high-power occasions, overload slippage plays a role in buffering and protecting the transmission device. On the other hand, the engagement drive relies on the engagement of the active and passive parts or intermediate parts to transfer power and motion, such as in gear and chain drives. The engagement drive can be used for high-power occasions. Although it has a good transmission accuracy, it requires high manufacturing and installation accuracies [12]. Most currently available commercial robotic hands adopt the gear drives, and some use direct drives to obtain the maximum transmission accuracy and transmission efficiency. The commercial robotic arm generally adopts harmonic drive to obtain high reduction ratio and high precision and also to reduce noise and vibration. However, underresearch robotic hands and arms often use a variety of drive methods because each drive method has advantages over other drive methods.

In this paper, the different drive methods used in commercial and underresearch humanoid robotic hands and arms are reviewed, and the specific application details of each drive method are summarized. Further, we compared the weight and output force of the robotic hands and arms with different drive methods, and we roughly summarized the influence of the drive system on the weight and output force. This study is aimed at analyzing and comparing the advantages and disadvantages of each drive method and discussing how to choose the appropriate drive method to exploit its strengths so that future researchers can consider the drive method used in humanoid robotic upper limbs.

The remainder of this paper is organized as follows.  Section 2 introduces the advantages and disadvantages of the main drive methods.  Section 3 introduces the anatomy of the human hand and the application of the main drive methods in humanoid robotic hands; further, the effect of different drive methods on weight and power output are also analyzed and discussed.  Section 4 introduces the anatomy of the human arm and the application of main drive methods in humanoid robotic arms; further, the effect of different drive methods on weight and payload are analyzed and discussed. Finally, Section 5 concludes the paper.

## 2. Main Drive Methods

### 2.1. Tendon Drive

The tendon drive uses metal, plastic, or nylon cables to simulate human tendons for motion and power transmission. Compared with other drive methods, the tendon drive has limitations in terms of precision, load, and durability; however, it has advantages in terms of miniaturization, lightness, and flexibility. Further, tendon drive systems enable actuators to be positioned at any desired location owing to the possibility of long-distance transmission.

However, additional transitions are required to route the tendon along the designed path. Currently, tendons are routed using sheaths, sliding surfaces, and pulleys [13], and the friction loss along the tendon is ranked from low to high for pulleys, sliding surfaces, and sheaths.

Tendon drive systems can be categorized into closed-loop and open-ended tendon drive systems [14]. A closed-loop tendon drive system comprises two tendons wound in opposite directions on two pulleys (actuator pulley and joint pulley). However, an open-ended tendon drive system contains only one tendon, and the other tendon is replaced by springs.

### 2.2. Gear Drive

The gear drive is used in a wide variety of modern equipment. The gear drive has the advantages of high transmission accuracy, high transmission efficiency, compact structure, reliable operation, and high durability. However, the requirements for gear installation are high, and they are not suitable for long-distance transmission. Further, the shock absorption and impact resistance are not as good as that of the belt drive and other flexible transmissions.

According to the difference in tooth shape, gears can be classified into spur, bevel, and worm gears, and each type has its own advantages and disadvantages; further, each type is suitable for different applications. Spur gears are the most widely used and easiest to install; they achieve larger reduction and torque ratios. Multistage reduction spur gear systems are commonly used in industrial equipment. Bevel gears can change the direction of transmission, and they have the characteristics of smooth transmission, low noise, and high load capacity. Worm gears have two advantages: it achieves a larger kinematic transmission ratio while requiring a minimum amount of space, and it exhibits self-locking properties [15].

The design of the gear drive system needs to consider the backlash between gears. If the backlash is too small, it will affect transmission efficiency; however, if the backlash is too large, it will affect transmission accuracy, while the tooth surface impact will produce vibration and noise, thereby affecting gear life.

### 2.3. Link Drive

The link drive connects components with each other via hinges or slides to realize motion and power transmission. The link drive can withstand large loads and achieve long-distance transmission. In addition, it can convert rotary motion to rotary or linear motion. However, the link mechanism must be driven through intermediate components, which are prone to large accumulation errors and low transmission efficiency.

A four-bar linkage, which is also called a four-bar, is the most common link drive mechanism. The four-rod mechanism can be divided into three basic forms based on whether the connecting rod can make a full circumference rotation, i.e., the crank rocker mechanism, double crank mechanism, and double rocker mechanism [16].

### 2.4. Fluid Drive

The fluid drive system can be classified into hydraulic drive system and pneumatic drive system based on the transmission medium. Compared to other drive systems, fluid drive systems have poorer transmission accuracy and the overall weight of the hydraulic drive system is larger, therefore they are less selected for robotic upper limbs.

The hydraulic drive transfers motion evenly and smoothly, and it can achieve overload protection. However, it is sensitive to changes in the external environment and requires separate energy. Compared to the hydraulic drive, the pneumatic drive has a faster action response, simpler structure, and better adaptability to the environment; however, the smoothness of movement is poor, and there is a large exhaust noise.

### 2.5. Other Drive Methods

The belt drive is similar to the tendon drive. It can achieve long-distance transmissions, it is stable, and it can cushion the vibration; however, its load capacity and durability are weak. Owing to the difference in shape, belts can be divided into round belts, V-belts, multigroove belts, and timing belts [17]. The timing belt has no slippage and runs at a constant speed, and it is often used to transfer direct motion for indexing or timing purposes.

The chain drive has characteristics of high transmission efficiency and high transmission power; however, the chain drive system is large and has high installation requirements. The screw drive has the advantages of high transmission efficiency, high transmission accuracy, smooth operation, and high reliability; however, the opposite screw should not be used for long-distance transmission, and the cost is higher.

## 3. Humanoid Robotic Hands

### 3.1. Anatomy of the Human Hand

The human hand has five fingers—index finger, middle finger, ring finger, little finger and thumb. The anatomy of the human hand reveals that it is composed primarily of bones; there are a total of 27 bones, which can be roughly divided into three categories: carpal bones, metacarpal bones, and phalanges [5]. Carpal bones are composed of eight bones, and they are responsible for the overall movement of the palm and fingers. Metacarpal bones comprise five bones that connect carpals and phalanges, and they support objects when grasping them. The remaining 14 bones are called phalanges, a thumb contains two phalanges, and the four fingers each contain three phalanges. These phalanges are the most important and complex parts of the human hand, and they are responsible for grasping objects and gesturing in daily activities [18, 19].

Parts of the bone connecting to the other bone are called joints. The joints in the human hand are divided into carpometacarpal (CMC) joints, metacarpal phalangeal (MCP) joints, and interphalangeal (IP) joints. The IP joints can be divided into proximal interphalangeal (PIP) joints and distal interphalangeal (DIP) joints [6]. The CMC and MCP joints have two DOFs, i.e., flexion/extension and abduction/adduction, respectively, while the IP joints have only one DOF, i.e., flexion/extension. The four fingers each contain the DIP, PIP, and MCP joints, while the thumb contains the IP, MCP, and CMC joints, and thus, each of the four fingers has three joints and four DOFs, and the thumb has three joints and five DOFs [20].

The grasping force of the human hand varies considerably based on the differences in innate and acquired training. Bretz et al. [21] summarized the hand and finger force values of 16 male subjects. Their results indicated that the average hand forces of the right and left hands were 551.2 N and 505.2 N, respectively. The finger forces of the little, ring, middle, and index and that of the thumb were 30.8 N, 37.9 N, 55.1 N, 56.7 N, and 107.7 N for the right hand and 28.4 N, 37 N, 53.7 N, 60.4 N, and 109.5 N for the left hand, respectively.

### 3.2. Main Drive Methods in Humanoid Robotic Hands

#### 3.2.1. Tendon Drive in Robotic Hands

The tendon drive has the characteristics of small size, light weight, and long-distance transmission; therefore, it is used in humanoid robotic hands [22]. Currently available tendon drive robotic hands can be categorized based on whether actuators are positioned on the hand into intrinsic actuation pattern (IAP), extrinsic actuation pattern (EAP), and hybrid actuation pattern (HAP) [23]. The IAP hand has the closest transmission distance, and therefore, the friction loss is the lowest. For the SPRING Hand [24], Pisa/IIT SoftHand 2 [25] and SMARTHAND [26] place actuators in the palm. Because these three robotic hands are underactuated hands, the number of actuators is 1, 2, and 4, respectively, and therefore, the size is still the same as that of a human hand. The EAP tendon drive robotic hand places actuators in the forearm. Indeed, an EAP hand has the farthest transmission distance, and therefore, it has the highest friction loss. However, the EAP allows the robotic hand to be smaller in size and weight, while more powerful actuators can be used. For example, the DIST-Hand [22, 27] places 20 DC motors with a 2 kg·cm output torque into a single-motor package outside the hand. The DEXMART Hand [28] employs 20 motors on the forearm, and therefore, the hand structure is simplified, which provides more space for sensor integration and a more anthropomorphic weight distribution. The HAP tendon drive robotic hand is equipped with actuators on both the forearm and hand. For example, the RoboRay Hand [29] has seven high payload motors in the forearm and five small motors in the palm for high-powered grasping and precise grasping. The bioinspired hand [20] used forearm-mounted motors to drive the MCP joints and palm-mounted motors to drive the coupled PIP and DIP joints. In general, IAP is chosen for better modularity of the robotic hands; EAP allows the use larger actuators for greater gripping force, while the remaining hand space can be spared to install more sensors; HAP is more suitable for the cases in which the convenience of installation and gripping force of different joints need to be considered.

When the number of actuators is less than the DOFs, the robotic system is called an underactuated system. Underactuated systems are often used in tendon drive robotic hands to reduce the number of actuators and simplify the structure and to allow adaptive grasping. For example, the RTR II hand [30] has three fingers; each of which uses only one tendon to operate three joints simultaneously. The force exerted on the tendon produces a corresponding moment in each joint axis that is proportional to the joint pulley radius. Setting the joint pulley radius and spring stiffness ensures that the joints move in the sequence of the MCP joint, PIP joint, and DIP joint to achieve adaptive grasping. Jing et al. [31] designed a five-finger prosthetic hand with only three actuators; however, they could achieve 13 grasping patterns.

#### 3.2.2. Gear Drive in Robotic Hands

The gear drive has high precision and can achieve large reduction ratios. It is therefore often used in robotic hands to obtain larger grasping forces and execute more accurate grasping tasks. The NTU hand [32, 33] has five fingers, and a multistage spur gear system forms the structure of the fingers. The gear system has a reduction ratio of approximately 100 : 1 at the middle and proximal finger segments, and a reduction ratio of approximately 1000 : 1 at the finger base segment. Therefore, the hand can grasp objects up to 1 kg. However, the hand is fully actuated with 17 DOFs driven by 17 actuators and the actuators, gears, potentiometers, and tactile sensors are integrated in each finger; therefore, the overall weight is large (1569 g). Hirano et al. [34] designed a five-finger robotic hand that also uses spur gears, and 67 gears made using a 3D printer. Meanwhile, two special gear mechanisms with different underactuated movements were proposed to allow the entire hand to be controlled using only six actuators for 15 joints, which resulted in lower cost and lighter weight (458 g). Collahuazo and Ordoñez [35] and Krausz et al. [36] designed hands with a set of bevel gears at MCP joints to convert motor rotation into internal flexion/extension of the fingers and thumb. The Tokyo-TECH 100 N Hand II [37] presents an improved force amplification drive that contains a turbine and worm gear. This mechanism can amplify torque at any joint while increasing the ROM of each joint and reducing the size of the hand. In general, the multistage spur gear system and worm gear can improve the transmission reduction ratio for greater gripping force. In order to improve the stability of the grip, the self-locking function of the worm gear can be considered. Due to the shape of the actuator, the actuator at the finger joint is generally arranged along the direction of the finger, so bevel gears can be an option to change the drive direction.

#### 3.2.3. Link Drive in Robotic Hands

The finger structure of the link drive robotic hand comprises the drive linkage, and therefore, the overall number of parts can be reduced. The TUAT/Karlsruhe hand [38] consists of links, and the link system consists of link plates and link rods. Because parts of the link plates are movable, the mechanism can automatically and uniformly adjust the grasping force by adjusting the rotation angle of the actuators according to the size and softness of the object. We previously discussed an underactuated tendon drive system and a link drive that can achieve underactuated control. The Keio Hand [39] can use a single actuator to drive 15 joints of the five fingers simultaneously. The hand uses a five-finger underactuated link mechanism that can envelop complex-shaped objects and adjust the grasping force distribution according to the size of the object. The principle of grasping an object is that the fingers start to move when the actuator provides the driving force, and when all fingers are in contact with the object, the link starts to rotate, which keeps the fingers moving until the object is enveloped by the fingers. The link drive for fingers can make good use of its structural features while providing better gripping stability. However, the design is complicated, and the movement is fixed after the design is finalized.

#### 3.2.4. Fluid Drive in Robotic Hands

Fluid actuators have a large output force per unit volume, and therefore, they are used for robotic hands that require a large grasping force. The fluid actuator has lower friction in the actuator itself. ARMAR's Hand [40] has 5 fingers and 11 joints, of which 8 are active and 3 are passive. All active joints are actuated by small flexible fluid actuators. As a result, the robotic hand has the maximum grasping force of 110 N. The ZJUT hand [41] has a flexible pneumatic actuator (FPA) placed at each joint to control the movement of the joint. Because the joint is driven directly by the FPA, the torque output is more accurate, and it reduces friction and vibration. The Vanderbilt hand [42] has 17 coupling DOFs and is driven by five pneumatic actuators. The pneumatic actuators are placed on the proximal side of the forearm, which allows for a larger arrangement. Therefore, a higher-stroke, larger-capacity cylinder is selected to provide forces of up to 40 N to the finger joint and 60 N to the thumb joint.

#### 3.2.5. Other Drive Methods in Robotic Hands

DLR-Hand II [15, 43, 44], DLR-HIT II [45], SPRING Hand [24], and Intrinsic Hand [46] use a belt drive, but only as an auxiliary drive method. Takaki and Omata [47] designed a robotic hand that uses a screw drive in the thumb. Chain drive systems are large and have high installation requirements, and therefore, they are not commonly used in humanoid robotic hands.

### 3.3. Effect of Different Drive Methods on Robotic Hands

**Figure 1:**
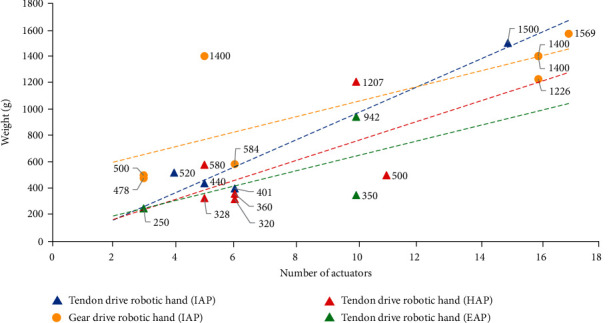
Comparison of the weight of humanoid robotic hands with different drive methods.

The robotic hand is located at the front of the arm, and therefore, a light weight can effectively reduce the inertial force. Meanwhile, if the robotic hand is used instead of a human prosthetic hand, the heavy weight will cause discomfort, and thus, the weight of the robotic hand needs to be minimized. The weight of a humanoid robotic hand contains the weight of the hand structure, actuator, and drive system. Because of the complexity of the robotic hand, the effect of a single factor on the overall weight of the hand is not known. Therefore, the remaining factors are unified to obtain the trend of the influence of a single factor on weight. Therefore, we compared the weights of the currently available five-finger, metal-based, IAP, HAP, and EAP tendons drive robotic hands and the IAP gear drive robotic hands based on the number of actuators and the drive method. The specifications of the robotic hands are summarized in Table 1, and the results of the comparison are shown in Figure 1.

The comparison results indicate that the overall weight of the robotic hand tends to increase as the number of actuators increases. Some tendon drive robotic hands weigh less than 400 g; however, all gear drive robotic hands weigh more than 400 g. This is because the tendons and pulleys of the tendon drive system are simple and lightweight compared to the gears of the gear drive system. Further, for the IAP robotic hands, when the number of actuators is small, the weight of the structure and drive system accounts for a larger proportion of the overall weight, but when the number of actuators is large, the weight of the actuators accounts for a larger proportion. Thus, as the number of actuators increases, the difference in weight between the tendon drive and gear drive robotic hands decreases. For the IAP, HAP, and EAP tendons drive robotic hands, as the number of actuators increases, the difference in weight increases.

The fingertip force of the available robotic hands is very different. There are robotic hands with a fingertip force of less than 5 N, such as Pisa/IIT SoftHand 2 [25], Gifu Hand I [66], Gifu Hand III [68], and CyberHand [55], and robotic hands with a fingertip force close to that of human fingers, such as Tokyo-TECH 100 N HAND [37]. The fingertip force is determined by the output torque of the actuator and the reduction ratio of the drive system. The fluid drive has higher energy output density and provides greater output torque for the same volume, while multistage gear drive systems can achieve a larger reduction ratio than other drive methods. They can be applied to robotic hands to increase gripping force.

## 4. Humanoid Robotic Arms

### 4.1. Anatomy of the Human Arm

The total weight of the human arm is approximately 5.2% of the body weight, of which the upper arm accounts for 3%, the forearm accounts for 1.6%, and the hands account for 0.6% of the body weight [69]. The human arm has seven DOFs, except for the hand. The shoulder joint is a ball-and-socket joint, with the anterior elevation (flexion) and posterior elevation (extension) of the upper arm centered on the medial and lateral axes of the joint. The lateral elevation of the upper arm (abduction) and movement towards the midline of a limb (adduction). The movement of twisting the upper arm outward (external rotation) and inward (internal rotation) around the upper arm. Anterior elevation (flexion) and posterior elevation (extension) of the upper arm are centered on the medial and lateral axes of the joint. Lateral elevation (abduction) of the upper arm is centered on the anterior-posterior axis, and the movement attracts the elevated upper arm to the trunk (adduction). The movement of twisting the upper arm outward (external rotation) and the movement of twisting the upper arm inward (internal rotation) around the upper arm are observed.

The elbow joint is a uniaxial joint that can only perform flexion and extension movements. In this case, there is only one movement axis, which runs horizontally across the elbow joint. In the forearm, the forearm bones—the radius and ulna—are arranged almost in parallel and form an axis. The forearm bone allows for twisting movements (pronation and supination) of the forearm. Meanwhile, the radius is shaped to move around the ulna. The wrist joint is a biaxial joint, which enables the bending and stretching movements of the wrist. These moves are relatively large. The movement of tilting sideways, i.e., the movement of abduction and adduction, are relatively small [70, 71].

### 4.2. Main Drive Methods in Humanoid Robotic Arms

#### 4.2.1. Tendon Drive in Robotic Arms

The actuator of a robotic arm is placed in the arm; however, because of the large distance of the center of mass (COM) from the shoulder base, a large inertial force is generated during movement. To minimize the inertial force, a tendon drive robotic arm is considered by placing the actuator as close as possible to the shoulder base. For example, the LIMS arm [72, 73] and the MYOROBOTICS arm [74] place the actuator on the shoulder joint. Thus, the COM of the LIMS arm is located only 169 mm from the shoulder base, and it has a reduced inertia force of 0.57 kg·m^2^, which is similar to the inertia force of the human arm, while the MYOROBOTICS arm has a lower inertia force of 0.2–0.4 kg·m^2^.

In an uncoupled drive system, in which one motor drives one joint separately, during the single-joint motion, all the other motors are in standby, which can be considered a waste of motor resources. However, a coupled drive system can reallocate motor resources to achieve greater payload. It is easier to use tendon drive implement coupled drive than the other drive methods due to the flexibility of its motor arrangement and wire routing.

A coupled tendon drive system is a drive system with two or more actuators working simultaneously during a single-joint motion. A coupled tendon drive system can reduce the size and weight of the structure by using smaller actuators with the same torque output conditions and output a larger joint torque with the same actuator. Coupled tendon drive systems are used for tendon drive robotic arms. For example, the CT Arm [75] was first applied with a coupled tendon drive, which is driven by six tendons to three joints, where tendon 1 and tendon 2 connect joint 1; tendon 3 and tendon 4 connect joints 1 and 2; and tendons 5 and 6 connect joints 1, 2, and 3. Li et al. [76, 77] designed a 7-DOF robotic arm with a modular coupled tendon drive system, with a 2-motor 2-DOF (2M2D) coupling drive module for the elbow joint and wrist joint, and a 3-motor 3-DOF (3M3D) coupling drive module for the shoulder joint. Here, the *n*-motor *n*-DOF coupling drive module represents *n* degrees of freedom that are coupled by *n* motors. Thus, the total weight of the robot arm is only 2.2 kg; however, the maximum weight that can be lifted is 1.5 kg.

#### 4.2.2. Gear Drive in Robotic Arms

A gear can produce a mechanical advantage through a gear ratio, and the geared devices can change the speed, torque, and direction of a power source. The Animator arm [78] uses a gearbox comprising multiple spur gears to change the rotational speed ratio and torque ratio of the input and output. Bennett et al. [79] designed a robotic arm with a set of worm gears in the wrist, thereby providing a 30 : 1 ratio in a smaller size. The 7R arm [80] has seven joints with a cylindrical gear used in joint 1, a double bevel gear used in joint 2, and a bevel gear used in joints 3 and 5. Both joints 1 and 2 use a 1.5 : 1 reduction ratio, and therefore, the maximum torque is greater than 60 Nm. The harmonic gear drive has the advantages of high transmission ratio, high transmission efficiency, high transmission accuracy and low noise, so it is widely used in commercial robotic arms.

#### 4.2.3. Fluid Drive in Robotic Arms

Kawashima et al. [81] designed a 6-DOF pneumatic robotic arm comprising a 2-DOF shoulder joint, 2-DOF elbow joint, and 2-DOF wrist joint. Each DOF is supported by two pneumatic artificial muscles (PARMs) for motion and power transmission. The ForceRobot arm [82] also uses a pneumatic drive with four pneumatic cylinders mounted on the shoulder. Because the maximum pressure of the air compressor used is 6.5 kgf/cm^2^, a maximum torque of 88 Nm can be generated in the shoulder.

#### 4.2.4. Other Drive Methods in Robotic Arms

The LIMS arm [73] employs a belt drive used in the shoulder to achieve an additional reduction ratio in addition to the reduction by the tension amplifying mechanism. Bennett et al. [79] designed a robotic arm with a three-stage transmission comprising two chain stages (ratio of 5.1 : 1 and 2.9 : 1, respectively) followed by a tendon drive output stage (with a ratio of 2.4 : 1) in the elbow. The chain drive is selected for the first two stages because of its high efficiency and compact nature. The arms for a collaborative robot [83] and an industrial robot [84] use four-bar linkages on elbow joints with a counterbalance mechanism (CBM).

### 4.3. Effect of Different Drive Methods in Robotic Arms

The main drive methods of the robotic arm are tendon and gear drives. The tendon drive has the advantages of light weight and long-distance transmission that allows the actuators to be arranged near the shoulder to reduce the inertia. The gear drive has the advantages of high transmission ratio, high transmission efficiency, and high transmission accuracy. Harmonic gears have the advantages of smaller size, lighter weight, and higher transmission ratio compared to conventional gears, thus allowing for greater output torque with less weight, as well as lower inertia forces.

**Figure 2:**
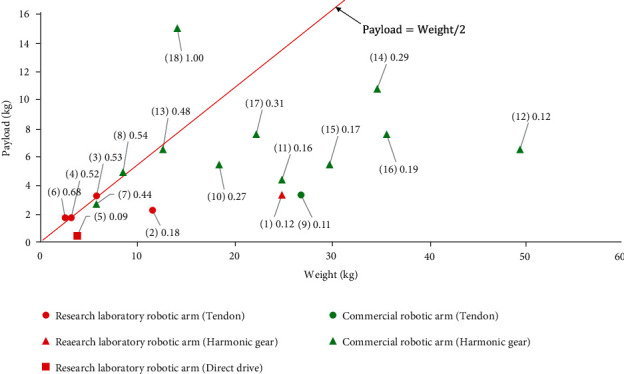
Comparison of the weight and payload of research robotic arms and commercial robotic arms with different drive methods, where the red line represents the payload weight as half of the arm's self-weight.

The weight of robotic arms affects their applications where lightweight robotic arms can be used as prosthetic arms for the disabled, whereas heavy robotic arms are used in factory production. Further, there is a relationship between the weight and the payload of the robotic arm, and theoretically, as the weight of the robot arm increases, the payload also increases. As a researcher, the main purpose is to make the payload capacity per unit weight as high as possible. The weight and payload of the research robotic arms and commercial robotic arms are summarized in Table 2, and the comparison of weight and payload is shown in Figure 2.

The results show that the ratio of the payload to the weight for general robots is less than 0.5, while that for better performing robotic arms is close to or slightly more than 0.5. The LWR III arm [94] has a self-weight of 14 kg and a maximum load of 14 kg, which achieves a load-to-weight ratio of 1. The use of harmonic drive gears of robust ILM motors with high power density and light materials and as a consequent light-weight-oriented mechanical design are key issues for reaching this goal. The LWH arm has the smallest payload capacity per unit weight, mainly because the robotic arm uses direct drive, and the motor torque is transferred to the joint in a 1 : 1 transmission ratio.

In order to increase the load capacity per unit weight of the robotic arm, two approaches can be considered. One is to reduce the weight of the robotic arm itself, such as the use of tendon drive, the use of lightweight materials; The other is to increase the output torque, which can generally be achieved by increasing the transmission ratio. Most commercial robotic arms use harmonic gear to obtain a high transmission ratio. In general, prosthetic arms are required to be as lightweight as possible to improve wearability. Therefore, the former approach is more applicable to the development of prostheses that reduce weight while ensuring the same load capacity. The use of tendon drive can well achieve the lightweight of the robotic arm by arranging the actuator close to the shoulder base to reduce the inertia. In addition, the elasticity of the tendons can improve the safety of the robotic arm to a certain extent. Industrial robotic arms are less demanding on lightweight than transmission accuracy and maximum payload. The latter approach is more suitable for industrial robotic arms.

## 5. Conclusions

**Figure 3:**
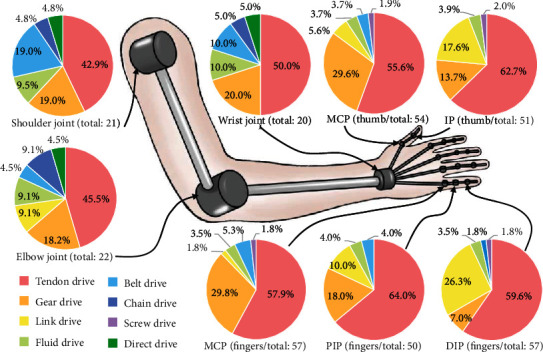
Proportion of each drive method used in humanoid robotic upper limb joints.

This study focuses on the application of different drive methods to humanoid robotic upper limbs. A statistical survey of the main drive methods used in the humanoid robotic upper limb joints showed that tendon drives are most commonly used in the robotic upper limb, gear drives are often used in the MCP joints of the thumb and fingers, and link drives are often used in the PIP and DIP joints of the fingers, the IP joint of the thumb, and the elbow joint; and finally, belt drives are widely used in the shoulder joint (shown in Figure 3). According to the structural characteristics of the robotic upper limb and the tasks to be performed, suitable drive methods are selected to ensure that the mechanism has a more desirable design output. A compound drive system comprising multiple drive methods considers the advantages of different drive methods and is widely used in robotic mechanisms. In addition, the reasonable use of the underdrive as well as the coupled drive can sometimes further optimize the robotic mechanism.

## Figures and Tables

**Table 1:** Specifications of humanoid robotic hands with different drive methods.

No.	Name	Fingers	Joints/DOFs/actuators	Actuation configuration	Transmission mechanism	Weight (g)	Ref.
(1)	SMARTHAND	5	16/16/4	DC motor/IAP	Tendon	520	[26]
(2)	Mitsui et al.	5	15/5/5	DC motor/ IAP	Tendon+pulley	440	[48]
(3)	Prosthetic hand	5	11/6/6	DC motor/IAP	Tendon+pulley+linkage+gear	401	[49]
(4)	DLR-HIT II	5	20/15/15	DC motor/IAP	Tendon+timing belt	1500	[50] [51]
(5)	Vanderbilt hand	5	16/16/5	DC motor+servomotor/HAP	Tendon+routing	580	[52]
(6)	Takaki & Omata	5	13/13/5	DC motor/HAP	Tendon+pulley+screw	328	[47]
(7)	RCH-1	5	16/16/6	DC motor/HAP	Tendon	320	[53] [54]
(8)	CyberHand	5	16/16/6	DC motor/HAP	Tendon+pulley	360	[55]
(9)	Bio-inspired	5	15/20/11	DC motor/HAP	Tendon+pulley+screw	500	[20]
(10)	EMG	5	15/15/10	Servomotor/HAP	Tendon +pulley	1207	[56]
(11)	Zollo et al.	5	15/16/5	DC motor/EAP	Tendon+pulley+worm gear+screw	330	[57]
(12)	Seki et al.	5	18/13/10	Servomotor/EAP	Tendon	350	[58]
(13)	Xu & Todorov	5	15/20/10	Servomotor/EAP	Tendon+pulley	942	[59]
(14)	Anthrobot-2	5	20/16/16	Servomotor/EAP	Tendon+pulley	794	[60] [61]
(15)	HIT/DLR	5	13/9/3	DC motor/IAP	Bevel gear+harmonic gear+linkage	500	[62] [63]
(16)	SSSA-MyHand	5	10/4/3	DC motor/IAP	Worm/spur/bevel gear+linkage	478	[64]
(17)	Koganezawa & Ito	5	15/10/5	DC motor/IAP	Planetary/worm gear+linkage+timing belt	1400	[65]
(18)	Krausz et al.	5	10/6/6	DC motor/IAP	Worm/spur/bevel gear+timing belt	584	[36]
(19)	Gifu Hand I	5	20/16/16	Servomotor/IAP	Bevel gear	1226	[66]
(20)	Gifu Hand II	5	20/16/16	Servomotor/IAP	Face gear	1400	[67]
(21)	Gifu Hand III	5	20/16/16	Servomotor/IAP	Face gear	1400	[68]
(22)	NTU	5	17/17/17	DC motor/IAP	Spur gear	1569	[32] [33]

**Table 2:** Specifications of humanoid robotic arms with different drive methods.

No.	Name	DOFs/actuators	Transmission mechanism	Weight (kg)	Payload (kg)	Payload/weight	Ref.
(1)	MIA	7/14	Harmonic gear	25.0	3.0	0.12	[85, 86]
(2)	Quigley et al.	7/7	Tendon + Timing Belt	11.4	2.0	0.18	[87]
(3)	LIMS	7/7	Tendon + Timing Belt	5.5	2.9	0.53	[72, 73]
(4)	Tsumaki et al.	7/8	Tendon	2.9	1.5	0.52	[88]
(5)	LWH	8/8	Direct drive	3.5	0.3	0.09	[89]
(6)	Li et al.	7/7	Tendon	2.2	1.5	0.68	[76, 77]
(7)	KINOVA Gen2	7/7	Harmonic gear	5.5	2.4	0.44	[90]
(8)	KINOVA Gen3	7/7	Harmonic gear	8.3	4.5	0.54	[91]
(9)	WAM Arm	7/7	Tendon	27.0	3.0	0.11	[92]
(10)	UR5	6/6	Harmonic gear	18.4	5.0	0.27	[93]
(11)	Barrett	7/7	Tendon	25.0	4.0	0.16	
(12)	KR Agilus 6 R700	6/6	Harmonic gear	50.0	6.0	0.12	
(13)	LWA Powerball	6/6	Harmonic gear	12.5	6.0	0.48	
(14)	LWAPA10	7/7	Harmonic gear	35.0	10.0	0.29	
(15)	SIA5F	7/7	Harmonic gear	30.0	5.0	0.17	
(16)	VS-6577G-B	6/6	Harmonic gear	36.0	7.0	0.19	
(17)	LBR iiwa7R800 7	7/7	Harmonic gear	22.3	7.0	0.31	
(18)	LWR III	7/7	Harmonic gear	14.0	14.0	1.00	[94]
